# Aggressive Angiomyxoma, Angiomyofibroblastoma, and Cellular Angiofibroma of the Lower Female Genital Tract: Related Entities With Different Outcomes

**DOI:** 10.7759/cureus.29250

**Published:** 2022-09-17

**Authors:** Saroona Haroon, Laraib Irshad, Shamail Zia, Abrahim H Ali, Tanim Ud Dowlah, Khushbakht Rashid, Umair Arshad Malik, Anam N Khan, Muhammad Irfan, Atif A Hashmi

**Affiliations:** 1 Pathology, King's Mill Hospital, Mansfield, GBR; 2 Pathology, Aga Khan University, Karachi, PAK; 3 Internal Medicine, Dow University of Health Sciences, Karachi, PAK; 4 Pathology, Ziauddin University, Karachi, PAK; 5 Internal Medicine, Bangladesh Medical College, Dhaka, BGD; 6 Internal Medicine, Liaquat National Hospital and Medical College, Karachi, PAK; 7 Internal Medicine, Aga Khan University, Karachi, PAK; 8 Pathology, Dow University of Health Sciences, Karachi, PAK; 9 Statistics, Liaquat National Hospital and Medical College, Karachi, PAK; 10 Pathology, Liaquat National Hospital and Medical College, Karachi, PAK

**Keywords:** mesenchymal tumors of lower female genital tract, female genital tract tumors, cellular angiofibroma, angiomyofibroblastoma, aggressive angiomyxoma

## Abstract

Introduction

Mesenchymal tumors of the lower female genital tract (FGT) are a miscellaneous group of tumors that include aggressive angiomyxoma (AAM), angiomyofibroblastoma (AMFB), cellular angiofibroma (CAF), and related entities. Histologically, these tumors are composed of stromal cells admixed with vessels, with some minor histological differences. An accurate diagnosis of these tumors is important owing to the differences in the outcome. In this study, we determined the clinicopathological characteristics of these tumors in our population and their association with recurrence.

Methods

This was a retrospective cross-sectional study conducted at the Department of Histopathology, Aga Khan University, from January 2005 to December 2019 over a period of 15 years. A total of 207 cases that were diagnosed as AAM, AMF, and CAF were selected for inclusion in the study. Clinical data, including age and location of the lesion, were obtained from histopathology referral forms. Tissue blocks of all cases were retrieved. Follow-up data were obtained from the patient files, and information regarding disease recurrence was recorded. One histological section from each tissue block was stained with hematoxylin and eosin stain, and histopathological findings were recorded. Additionally, immunohistochemical (IHC) studies, including vimentin, smooth muscle actin (SMA), and desmin were conducted on representative tissue blocks. Final histopathological diagnoses were rendered considering clinical, histopathological, and IHC findings.

Results

The median age of patients involved in the study was 33 years and the median tumor size was 5 cm with a predilection for the vulva (47.3%). AAM showed a predilection for patients between the ages of 31-45 years, while AMFBs and CAFs were most common in younger age groups of less than 30 years. In 46.8% of cases, the tumor size of AAM was between 6 and 10 cm, while in all cases of CAF (100%) and the majority of AMFB cases (53.2%) the tumor size was smaller than 5 cm. Histologically, in all cases of AAM, the lesional cells were spindle (100%), whereas, in 13% of cases epitheloid cells were observed, with myxoid stroma in 92.2% cases. The presence of stromal smooth muscle was noted in 42.9% of cases, in 79.2% of cases the vessels were thick-walled, with 54.5% having hyalinized vessels, and most of the cases (77.9%) had ill-defined borders. Among IHC findings, AMFB was most frequently positive for actin (62.2%), while AAM and AMFB showed more frequent staining for desmin compared to CAF. A significantly higher recurrence rate was observed in AAM (27.3%), compared to AMFB and CAF. A significant association of recurrence was seen with tumor size. It was noted that the recurrence rate was directly proportional to the size of the tumor and was highest (60%) with a tumor size of more than 10 cm.

Conclusion

In our study, we noted that AMFB was the most common among the three mesenchymal tumors of the lower FGT. In contrast, AAM had the highest recurrence rate, and recurrence was significantly associated with tumor size. Histological findings, especially the type of stromal cells and background vasculature, are of utmost importance for the correct recognition of these tumors, while the role of IHC studies is limited.

## Introduction

Mesenchymal tumors of the lower female genital tract (FGT), especially involving the vulva, are a miscellaneous group of tumors that include aggressive angiomyxoma (AAM), angiomyofibroblastoma (AMFB), cellular angiofibroma (CAF) and related entities. Histologically, these tumors are composed of spindle to epithelioid stromal cells admixed with vessels, with subtle histological differences. An accurate diagnosis of these tumors is important owing to the differences in the outcome and management. AAMs are relatively aggressive mesenchymal lesions unique to the lower FGT, pelvis and perineum characterized grossly by large size (mostly >10cm) and ill-circumscribed borders. Microscopically, they are composed of spindle cells embedded in hypocellular myxoid stroma, and thick-walled blood vessels [1,2]. AMFBs are related to AAM, however, they are clinically indolent and grossly circumscribed. Microscopically, they show alternating hypo and hypercellularity, and are composed of spindle to epithelioid lesional cells. Characteristic thick-walled muscular vessels are seldom seen in AMFB, as opposed to AAM [3,4]. Similar to AMFB, CAFs are benign tumors of the vulvovaginal region with a very low recurrence rate. Histologically, they show spindle cells with collagenized to hyalinized stroma and hyalinized medium-size blood vessels [5,6]. Other rare mesenchymal tumors of this region include leiomyomas, superficial angiomyxomas, fibroepithelial polyps, and low-grade fibromyxoid sarcomas. The relative frequency of these tumors in our population is unknown. In this study, we determined the clinicopathological characteristics of these tumors in our population.

## Materials and methods

This is a retrospective cross-sectional study conducted at the Department of Histopathology, Aga Khan University, from January 2005 to December 2019 over a period of 15 years. Cases that were diagnosed as AAM, AMFB, and CAF were selected for inclusion in the study. Cases of lower FGT tumors other than AAM, AMFB, and CAF, like fibroepithelial polyps and superficial angiomyxomas, were excluded from the study. Clinical data, including age and location of the lesion, were obtained from histopathology referral forms. Tissue blocks of all cases were retrieved. Follow-up data were obtained from the patient files, and information regarding disease recurrence was recorded. Cases with missing tissue blocks or clinical/follow-up data were excluded from the study. A total of 207 cases met the inclusion/exclusion criteria and were thus included in the study.

All specimens were resection specimens of lower FGT tumors. Tru-cut and incisional biopsies were excluded from the study. The resected specimens were received by the histopathology laboratory in 10% buffered formalin. After formalin fixation, grossing of the specimens was done according to standard protocols. Tumor size was recorded and sections from resection margins were submitted. Additionally, one representative section per centimeter of the maximum tumor dimension was submitted, followed by routine tissue processing. Cases with incompletely resected tumors (tumors with positive margins) were excluded from the study. 

One histological section from each tissue block was stained with hematoxylin and eosin stain, and histopathological findings, including the type of lesional cells (spindled vs. epithelioid), stromal characteristics (myxoid, collagenized, or hyalinized), and background vessels (thick-walled muscular vessels, collagenized vessels) were recorded. Additionally, immunohistochemical (IHC) studies, including vimentin, smooth muscle actin (SMA), and desmin were conducted on representative tissue blocks. Final histopathological diagnoses were rendered considering clinical, histopathological, and IHC findings.

Data analysis was performed using SPSS Statistics v. 26.0 (IBM Corp., Armonk, NY, USA). Chi-square, Fisher’s exact, and Kruskal-Wallis H tests were used to verify the association. Survival analysis was done by the Kaplan-Meier method. P-values <0.05 were considered to be significant.

## Results

Our study demonstrated that the mesenchymal tumors of the FGT showed a wide age distribution with the peak incidence between the age of 31-45 years in 42% of cases, followed by patients of <30 years of age (38.6%), with the median age of 33 years. Grossly, the median tumor size in our study was 5 cm. In 47.3% of cases, the tumor size was <5 cm, whereas in only 15% of cases the tumors were more than 10 cm. It was noted that tumors showed a marked predilection for the vulva in 47.3%, followed by the vagina (27.5%) and labia majora (25.1%). The histological findings in our study showed that the lesional cells were spindle in 58.5% cases and epithelioid in 52.5% cases, surrounded by collagenized stroma in 63.3% cases, myxoid in 41.1%, and hyalinized in 31.9% cases; the vasculature being composed of hyalinized vessels in 50.7% cases and thickened muscular vessels in 41.5% of the cases. Well-defined borders were present in 57.5% of cases. The IHC findings revealed that the background stroma stained for vimentin in most cases (92.3%), while SMA was positive in 54.1% of cases and desmin in 28% of cases. In our study, AMFB (53.6%) was the most frequent diagnosis, followed by AAM (37.2%), and CAF (9.2%). The overall recurrence rate of these tumors was found to be 14.5%. The median time interval between diagnosis and recurrence was four years (interquartile range [IQR]: 3) (Table 1).

**Table 1:** Clinicopathological features of the population under study IQR: Interquartile range

Clinicopathological features	Values
Age (years)	
Median (IQR)	33.0 (15)
Age groups	
≤30 years, n (%)	80 (38.6)
31-45 years, n (%)	87 (42)
>45 years, n (%)	40 (19.3)
Tumor size (cm)	
Median (IQR)	5.0 (4.0)
Tumor size groups	
<5 cm, n (%)	98 (47.3)
6-10 cm, n (%)	78 (37.7)
>10 cm, n (%)	31 (15)
Follow-up duration (years), median (IQR)	8.0 (5.0)
Tumor Site	
Labia majora, n (%)	52 (25.1)
Vagina, n (%)	57 (27.5)
Vulva, n (%)	98 (47.3)
Histological findings	
Lesional cells	
Spindle	
Yes, n (%)	121 (58.5)
No, n (%)	86 (41.5)
Epithelioid	
Yes, n (%)	108 (52.2)
No, n (%)	99 (47.8)
Stroma	
Myxoid stroma	
Yes, n (%)	85 (41.1)
No, n (%)	122 (58.9)
Collagenized stroma	
Yes, n (%)	131 (63.3)
No, n (%)	76 (36.7)
Hyalinized stroma	
Yes, n (%)	68 (31.9)
No, n (%)	141 (68.1)
Stromal smooth muscles	
Yes, n (%)	46 (22.2)
No, n (%)	161 (77.8)
Vasculature	
Thickened muscular vessels*	
Yes, n (%)	86 (41.5)
No, n (%)	121 (58.5)
Hyalinized vessels	
Yes, n (%)	105 (50.7)
No, n (%)	102 (49.3)
Borders	
Ill defined, n (%)	88 (42.5)
Well defined, n (%)	119 (57.5)
Immunochemical findings	
Smooth muscle actin	
Positive, n (%)	112 (54.1)
Negative, n (%)	95 (45.9)
Vimentin	
Positive, n (%)	191 (92.3)
Negative, n (%)	16 (7.7)
Desmin	
Positive, n (%)	58 (28)
Negative, n (%)	149 (72)
Diagnosis	
Aggressive angiomyxoma, n (%)	77 (37.2)
Angiomyofibroblastoma, n (%)	111 (53.6)
Cellular angiofibroma, n (%)	19 (9.2)
Recurrence	
Yes, n (%)	30 (14.5)
No, n (%)	177 (85.5)

Table 2 demonstrates the association of the tumors with clinicopathological findings. AAM showed a predilection for patients between the ages of 31-45 years, whereas AMFBs and CAFs were most common in younger age groups of less than 30 years. In 46.8% of cases, the tumor size of AAM was between 6 and 10 cm, while in all cases of CAF (100%) and in most cases of AMFB (53.2%) the tumor size was <5 cm. In our study, all three tumors, AAM (54.5%), AMFB (34.2%), and CAF (94.7%) showed a predilection for the vulva; however, CAF was infrequently seen at the other two sites (vagina and labia majora). Histologically, in all cases of AAM, the lesional cells were spindle (100%), whereas, in 13% of cases epitheloid cells were observed, with myxoid stroma in 92.2% cases, the presence of stromal smooth muscle was noted in 42.9% cases. In 79.2% of cases, the vessels were thick-walled, with 54.5% having hyalinized vessels, and most of the cases (77.9%) had ill-defined borders. Among IHC findings, AMFB was most frequently positive for actin (62.2%), while AAM and AMFB showed more frequent staining for desmin compared to CAF. A significantly higher recurrence rate was observed in AAM (27.3%), compared with AMFB and CAF. Histologically, AMFBs were predominantly composed of epitheloid cells (85.6%), surrounded by collagenized stroma in most cases (80.2%). Around 89.2% of cases of AMFBs had well-defined borders with a recurrence rate of 7.2%. Immunohistochemically, these tumors stained positive for vimentin in most cases (92.8%), while SMA staining was seen in 62.2% of cases. Histological findings of CAF showed that the lesional cells were spindle with hyalinized stroma in most cases (84.2%). The IHC findings showed that CAFs stained positive for vimentin in 84.2% of the cases. The recurrence rate of CAFs in our study was found to be the least at only 5.3%.

**Table 2:** Association of clinicopathological features with the diagnosis IQR:  Interquartile range *Kruskal-Wallis H test was applied, **Fisher exact/Chi-square test was applied, ***Significant as < 0.05

Clinicopathological features	Diagnosis			p-value
	Aggressive angiomyxoma	Angiomyofibroblastoma	Cellular angiofibroma	
Age (years)				
Median (IQR)*	42.0 (13.5)	32.0 (13.0)	28.0 (13.0)	0.001***
Age groups**				
≤30 years, n (%)	18 (23.4)	49 (44.1)	13 (68.4)	0.003***
31-45 years, n (%)	40 (51.9)	43 (38.7)	4 (21.1)	
>45 years, n (%)	19 (24.7)	19 (17.1)	2 (10.5)	
Tumor size (cm)				
Median (IQR)*	7.0 (7.00)	4.0 (3.00)	2.0 (1.7)	<0.001***
Tumor size groups**				
<5 cm, n (%)	20 (26)	59 (53.2)	19 (100)	<0.001***
6-10 cm, n (%)	36 (46.8)	42 (37.8)	0 (0)	
>10 cm, n (%)	21 (27.3)	10 (9)	0 (0)	
Follow-up duration (years), median (IQR)*	8.0 (4.0)	7.0 (6.0)	8.0 (5.0)	0.193
Tumor Site**				
Labia majora, n (%)	16 (20.8)	36 (32.4)	0 (0)	<0.001***
Vagina, n (%)	19 (24.7)	37 (33.3)	1 (5.3)	
Vulva, n (%)	42 (54.5)	38 (34.2)	18 (94.7)	
Histological findings				
Lesional cells				
Spindle**				
Yes, n (%)	77 (100)	25 (22.5)	19 (100)	<0.001***
No, n (%)	0 (0)	86 (77.5)	0 (0)	
Epithelioid**				
Yes, n (%)	10 (13)	95 (85.6)	3 (15.8)	<0.001***
No, n (%)	67 (87)	16 (14.4)	16 (84.2)	
Stroma				
Myxoid stroma**				
Yes, n (%)	71 (92.2)	12 (10.8)	2 (10.5)	<0.001***
No, n (%)	6 (7.8)	99 (89.2)	17 (89.5)	
Collagenized stroma**				
Yes, n (%)	31 (40.3)	89 (80.2)	11 (57.9)	<0.001***
No, n (%)	46 (59.7)	22 (19.8)	8 (42.1)	
Hyalinized stroma**				
Yes, n (%)	17 (22.1)	33 (29.7)	16 (84.2)	<0.001***
No, n (%)	60 (77.9)	78 (70.3)	3 (15.8)	
Stromal smooth muscles**				
Yes, n (%)	33 (42.9)	11 (9.9)	2 (10.5)	<0.001***
No, n (%)	44 (57.1)	100 (90.1)	17 (89.5)	
Vasculature				
Thickened muscular vessels**				
Yes, n (%)	61 (79.2)	23 (20.7)	2 (10.5)	<0.001***
No, n (%)	16 (20.8)	88 (79.3)	17 (89.5)	
Hyalinized vessels**				
Yes, n (%)	42 (54.5)	48 (43.2)	15 (78.9)	0.011***
No, n (%)	35 (45.5)	63 (56.8)	4 (21.1)	
Borders**				
Ill-defined, n (%)	60 (77.9)	12 (10.8)	16 (84.2)	<0.001***
Well defined, n (%)	17 (22.1)	99 (89.2)	3 (15.8)	
Immunochemical findings				
Smooth muscle actin**				
Positive, n (%)	34 (44.2)	69 (62.2)	9 (47.4)	0.042***
Negative, n (%)	43 (55.8)	42 ( 37.8)	10 (52.6)	
Vimentin**				
Positive, n (%)	72 (93.5)	103 (92.8)	16 (84.2)	0.360
Negative, n (%)	5 (6.5)	8 (7.2)	3 (15.8)	
Desmin**				
Positive, n (%)	19 (24.7)	38 (34.2)	1 (5.3)	0.024***
Negative, n (%)	58 (75.3)	73 (65.8)	18 (94.7)	
Recurrence**				
Yes, n (%)	21 (27.3)	8 (7.2)	1 (5.3)	<0.001***
No, n (%)	56 (72.7)	103 (92.8)	18 (94.7)	

Table 3 shows the association of recurrence with clinicopathological characteristics. A significant association of recurrence was seen with tumor size. It was noted that the recurrence rate was directly proportional to the size of the tumor and was highest (60%) with a tumor size of more than 10 cm.

**Table 3:** Association of clinicopathological features with recurrence IQR: Interquartile range *Kruskal-Wallis H test was applied, **Fisher exact/Chi-square test was applied, ***Significant as < 0.05

Clinico-pathological features	Recurrence		
	Yes	No	p-value
Age (years)			
Median (IQR)*	42.0 (17.25)	32.0 (14.50)	0.015***
Age groups**			
≤30 years, n (%)	6 (20)	74 (41.8)	0.056
31-45 years, n (%)	15 (50)	72 (40.7)	
>45 years, n (%)	9 (30)	31 (17.5)	
Tumor size (cm)			
Median (IQR)*	13.0 (9.50)	4.0 (4.0)	0.184
Tumor size groups**			
<5 cm, n (%)	3 (10)	95 (53.7)	<0.001***
6-10 cm, n (%)	9 (30)	69 (39)	
>10 cm, n (%)	18 (60)	13 (7.3)	
Follow-up duration (years), median (IQR)*	8.5 (5.0)	8.0 (6.0)	0.195
Tumor site**			
Labia majora, n (%)	7 (23.3)	45 (25.4)	0.939
Vagina, n (%)	9 (30)	48 (27.1)	
Vulva, n (%)	14 (46.7)	84 (47.5)	
Smooth muscle actin**			
Positive, n (%)	14 (46.7)	98 (55.4)	0.377
Negative, n (%)	16 (53.3)	79 (44.6)	
Vimentin**			
Positive, n (%)	28 (93.3)	163 (92.1)	1.0
Negative, n (%)	2 (6.7)	14 (7.9)	
Desmin**			
Positive, n (%)	7(23.3)	51 (28.8)	0.537
Negative, n (%)	23 (76.7)	126 (71.2)	

**Figure 1:**
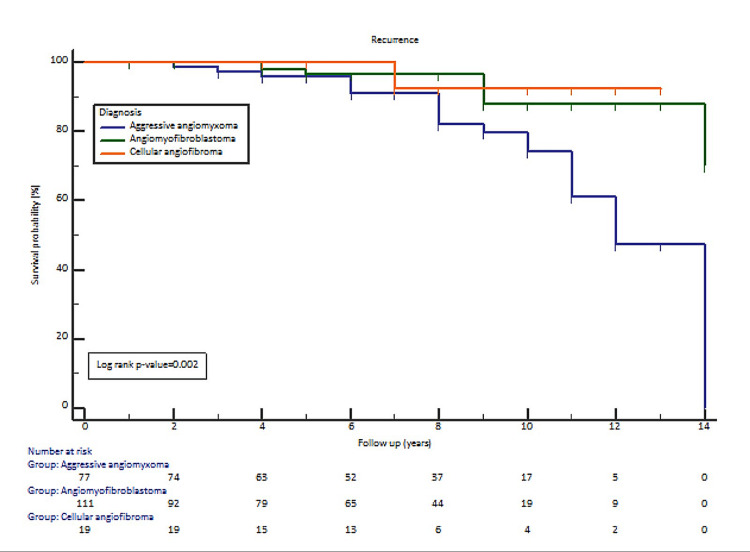
Survival analysis by Kaplan–Meier method

Figure 1 shows survival analysis by the Kaplan-Meier method. Recurrence was found to be significantly high in AAM, compared to AMFB and CAF (p=0.002).

## Discussion

Vulvo-vaginal mesenchymal neoplasms that are composed of spindle cells, most commonly arising in the vulvovaginal region, often pose diagnostic difficulties for pathologists due to their overlapping histological features [7]. We conducted a study on the three most common mesenchymal tumors of FGT, namely, AAM, AMFB, and CAF. We found that these tumors mostly involve patients between the ages of 31 and 45 years, with the vulva being the most common site, and most of the tumors were found to be of <5 cm in size. AMFB was the most frequent diagnosis, and AAM had the highest frequency of recurrence. Tumor size was significantly found to associate with the recurrence rate. The role of IHC findings was limited in the diagnosis of these tumors and histological characteristics were more diagnostic.

Aggressive angiomyxoma

AAM is a rare mesenchymal tumor of vulvo-vagina that is slow-growing with a high rate of local recurrence but a low metastatic rate [8]. It is a benign tumor that is locally aggressive [9].

We studied the histological and IHC features of these tumors and found that AAMs were all composed of spindle cells (100%) with epitheloid cells present in 13% of cases as well as embedded in a myxoid stroma in most cases; in a few cases stromal smooth muscle presence was noted. A prominent vascular component is a hallmark of this tumor type, including thickened muscular walls in 79.2% of cases and hyalinized vessels in 54.5% of cases. The IHC findings revealed that AAMs stained positive for vimentin in 93.5% of cases, SMA (44.2%), and less commonly for desmin (24.7%). In our study, we found AAM had the highest recurrence rate (27.3%). AAM showed a predilection for patients between the ages of 31 and 45 years. In 46.8% of cases, the tumor size of AAM was between 6 and 10 cm and most commonly affected the vulva, followed by the vagina. Fetsch et al. conducted a study on 29 female patients with AAM; similarly to our study they concluded that the peak incidence of AAM among their patients was in the fourth decade of life with the pelvis, perineum, and vulva being commonly affected sites with most of the tumors being 10 cm or more in size; histologically they found that the neoplastic cells were spindle to stellate having collagenized matrix with blood vessels of variable caliber, with few cells showing smooth muscle differentiation. Immunohistochemically, the neoplastic cells stained positive for desmin, SMA, and vimentin. They found AAM to have a high rate of local recurrence [10]. In another study conducted by Steeper et al. on the AAM of the female pelvis and perineum, they included nine cases of soft tissue tumors of the female pelvis and perineum. Similar to our study they found that the peak incidence of AAM in their subjects was between the age of 21-38 years and the tumors were of large sizes. Histologically, the tumor cells were mostly composed of spindle, and stellate cells were embedded in a loose myxoid stroma having larger vessels with thickened walls; these tumors were found to be locally infiltrative and recurrent [1].

Chen et al. evaluated the clinicopathological features of AAM in five cases having a median age of 42 years, and most cases affected the pelvis (3/5), whereas one each involved the vulva and buttock. The average size of these tumors was 18 cm and had ill-defined borders. Morphologically, they found that the lesional cells were spindle and stellate with myxedematous stroma containing vessels that were unevenly thickened and randomly arranged in the matrix. The IHC studies showed that the tumor cells were mostly positive for vimentin, desmin, estrogen receptors (ER) and progesterone receptors (PR), while weak expression of SMA was also noticed. They found that AAM had a high local recurrence range between 25 and 47% [11]. Due to the locally aggressive nature of AAM, these tumors should be managed appropriately and long-term follow-up of the patients should be done [12].

Angiomyofibroblastoma

AMFB is a rare, slow-growing mesenchymal tumor, with a predilection for FGT with a low tendency to reoccur. The majority of these tumors are small, measuring <5 cm [13]. The average age of occurrence of AFMB is 45.8 years [14]. In our study, we found that AMFBs had a predilection for the younger age group of <30 years of age, most commonly arising in the vulva, with a tumor size of <5 cm; these tumors were mostly composed of epitheloid cells in 85.6% of cases, with background collagenized stroma in 80.2%, though a few cases showed a variation of myxoid and hyalinized stroma. These neoplasms showed a prominent vascular component including hyalinized vessels. The IHC findings revealed that the stromal cells expressed immunoreactivity for vimentin in 92.8% of cases, whereas 34.2% of cases stained positively for desmin.

Magro et al. assessed the morphological and IHC features of 11 cases of vulvo-vaginal AMFB and concluded that the common histological features among the cases were the presence of spindle to epitheloid lesional cells embedded in an edematous to fibrous stroma having thin-walled vessels. All these tumors stained positive for vimentin, whereas in a few cases, tumor cells were positive for desmin and SMA [4]. Seo et al. reported a case of AMFB of the vulva, similar to our study; they found that the tumor cells expressed positive for vimentin and had estrogen/progesterone receptor expression [15].

Cellular angiofibroma

CAF is a rare benign mesenchymal tumor, which is fibroblastic, usually arising in the vulvovaginal area of middle-aged women, these tumors are small, usually <3 cm [16]. Typically, CAF is composed of spindle shape cells embedded in a stroma containing wispy collagen having medium-sized hyalinized vessels [17].

According to our study, CAF mostly occurred in the younger age group (<30cm), and the size of the tumor in most cases was <5 cm, showing a predilection for the vulvovaginal region, we found that the microscopic findings of CAF included spindle-shaped cells with intermixed epitheloid cells in a few cases, embedded in a hyalinized stroma in most cases (84.2%), whereas, in 57.9% of cases there was overlapping collagenized stroma, and the vascular component consisted of hyalinized blood vessels. Immunohistochemically, these tumors were positive for vimentin (84.2%) in most cases, followed by actin in 47.4% of cases.

Colombat et al. presented a case report of vulvar CAF in a 37-year-old female and found that morphologically the tumor cells were composed of spindle cells, with numerous thick and thin-walled blood vessels which were mostly hyalinized and the stromal cells showed immunoreactivity to vimentin [18]. Angelico et al. did an overview of vulvo-vaginal stromal tumors and concluded that CAF is composed of spindle cells with a predominant fibrous stroma, containing numerous hyalinized small-medium blood vessels [19]. Iwasa et al. studied the clinicopathological and IHC study of CAF in 51 cases and similar to our study, they found that the most common site of CAF among females was the vulvovaginal region. Microscopic studies of the tumor showed that these neoplasms were composed of spindle cells with an edematous to fibrous background stroma around small to medium-sized hyalinized vessels. Moreover, they found that most of the tumors (60%) expressed positively for CD34 [5].

Our study can be viewed with a few limitations, the most important of which is the lack of molecular analysis of these tumors. Therefore, we suggest that a detailed molecular analysis of these tumors should be carried out and new biomarkers should be sought that predict recurrence in these tumors.

## Conclusions

In this study, we found that AMFB is the most common mesenchymal tumor of the lower FGT, whereas AAM was more frequently associated with recurrence. We also noted that size is the most important factor in predicting recurrence in these tumors. Moreover, histological features of the background stroma and vasculature are pivotal in the exact characterization of these tumors, while the role of IHC studies is still limited. More studies are needed to identify prognostic biomarkers in these tumors, especially AAM, and to uncover the molecular features of these tumors.
